# The Role of Baby-Friendly Designated Hospitals in Breastfeeding Initiation Across Racial/Ethnic Groups in Florida

**DOI:** 10.1007/s10995-024-04011-4

**Published:** 2024-11-02

**Authors:** Cynthia N. Lebron, Michaela Larson, Jennifer Chavez, Alexa Parra

**Affiliations:** 1 School of Nursing and Health Studies, University of Miami, 5030 Brunson Dr, Coral Gables, FL USA; 2 Miller School of Medicine, University of Miami, Coral Gables, FL USA

**Keywords:** Breastfeeding, Baby-friendly hospital, Health disparities, Health equity, Minority health

## Abstract

**Background:**

Baby-Friendly Hospitals (BFH) in the United States (U.S.) are associated with higher breastfeeding initiation rates. Breastfeeding is associated with a myriad of favorable health outcomes for both mother and child. However, few studies have examined the impact of breastfeeding support resources, like BFH, on breastfeeding initiation among minority groups. The objective of this study is to evaluate the association between birth at a BFH and the breastfeeding initiation in Florida.

**Methods:**

A retrospective exploratory analysis of BFH and birth certificate data (n=3,321,022 ) from 2004-2022 from Florida was conducted.  A logistic regression model was fit to examine the main and interaction effects of race/ethnicity and birth at a BFH on breastfeeding initiation. Time was included as a sequential variable to adjust for temporal effects. Covariates known to impact breastfeeding initiation rates, including maternal education and prenatal care utilization, were included in multivariate analyses.

**Results:**

Of births at a BFH, 89% of mothers initiated breastfeeding. Comparatively, of the births at a non-BFH, 84% of mothers initiated breastfeeding. Giving birth at a BFH increased the odds of breastfeeding initiation by at least 42% (OR = 1.42, CI: 1.38-1.45, p <0.001, Hispanic White mothers) in unadjusted models and 10% (OR = 1.10, CI: 1.03-1.17, p = 0.004, other non-Hispanic mothers) in adjusted models. However, BFH may have differential effects by maternal race and ethnicity. In the multivariate model adjusting for relevant covariates, non-Hispanic Black mothers who gave birth at a BFH were 27% less likely to initiate breastfeeding compared to mothers that gave birth at a non-BFH (OR = 0.73, CI: 0.61- 0.88, p < 0.001; interaction term for BFH*maternal race/ethnicity). Similar trends were observed for Hispanic Black, Hispanic White, and other non-Hispanic mothers.

**Conclusions:**

Giving birth at a BFH is associated with greater odds of breastfeeding initiation. However, when considering the race and ethnicity of mothers, these odds significantly decline, indicating a need to further explore the barriers that may preclude non-Hispanic Black and Hispanic moms from receiving the same benefits of BFH.

## Significance

What We Already Know

What This Study Adds

Despite the known benefits, breastfeeding rates in the U.S. remain below optimal levels, especially among racial and ethnic minority populations. Initiatives like the Baby-Friendly Hospital Initiative (BFH) have been shown to improve breastfeeding initiation and exclusivity rates, but racial and ethnic disparities persist.

This study finds that while BFH hospitals are associated with increased breastfeeding initiation overall, minority mothers—particularly non-Hispanic Black mothers—experience lower breastfeeding initiation rates compared to their non-Hispanic White counterparts, even at BFH hospitals. The study highlights the need for culturally competent and racially conscious breastfeeding support to close the breastfeeding gap and improve health outcomes for minority populations.

## Introduction

Breastfeeding is shown to be an exceptional primary prevention intervention for infants and mothers alike (Gartner et al., 2005). The “personalised medicine provided by human milk (p.6)” (Victora et al., 2016) protects children against negative health outcomes such as otitis media, dental malocclusions, asthma, sudden infant death syndrome, and obesity (Chung et al., 2007). For nursing women, breastfeeding reduces the risk of breast cancer, improves birth spacing, and may also reduce the risk of ovarian cancer and diabetes (Victora et al., 2016). The American Academy of Pediatrics (AAP) recommends that infants be exclusively breastfed, or consume only mother’s milk and vitamin/mineral drops, for the first six months of life, with continued breastfeeding alongside the introduction of complementary foods for at least one year (Gartner et al., 2005). Despite these recommendations, data from the Centers for Disease Control and Prevention’s (CDC) 2022 Breastfeeding Report Card indicates that 43% of infants in the United States (U.S.) are exclusively breastfed through three months and 24.9% are exclusively breastfed through six months (Control & Prevention, 2022). Additionally, persistent racial and ethnic disparities in breastfeeding initiation, or receiving any breastmilk during the period between birth and discharge, duration and exclusivity exist. Non-Hispanic (NH) Black women continue to have the lowest rates of breastfeeding initiation and continuation at 6 months and 12 months compared with all other racial/ethnic groups in the U.S. (National Immunization Survey, 2019). Moreover, among breastfed infants, an average of 24% are introduced to formula by the second day of life, with higher rates in Hispanic (33%) and NH Black (32%) infants (Chapman & Pérez-Escamilla, 2012). The consequences of poor breastfeeding practices are substantial, especially for minority mothers and children. Many of the conditions against which breastfeeding protects disproportionately affect minority populations (Chapman & Pérez-Escamilla, 2012). For instance, NH Black infants experience a higher prevalence of infant mortality compared with other racial/ethnic groups (e.g., NH White infants)(Ely & Driscoll, 2020), however exclusive breastfeeding protects against infant mortality (Sankar et al., 2015). Additionally, breastfeeding protects against obesity (Baidal et al., 2016) and Hispanic children in every age group have the highest rates of obesity compared with other racial/ethnic groups (Ogden et al., 2020).

Minority women are more likely than NH White women to have a lack of social support, inadequate access to breastfeeding resources, and racially biased healthcare, all of which are shown to reduce the likelihood of exclusive breastfeeding (Johnson et al., 2015; Jones et al., 2015; Williams & Rucker, 2000). The Surgeon General’s Call to Action to Support Breastfeeding states that hospital practices and policies in maternity settings can undermine maternal and infant health by creating barriers to supporting a mother’s decision to breastfeed (U. D. o. Health & Services, 2011). Intentional, unintentional, and implicit biases, including providers having lowered expectations of their patients based on race, ethnicity, and socioeconomic status, are associated with receiving an overall lower standard of care and may influence breastfeeding rates (Robinson et al., 2019). These biases are especially poignant considering that mothers who believe that obstetric and pediatric care providers favor exclusive breastfeeding during the neonatal period had higher odds of exclusive breastfeeding than those who perceived providers’ preference to be neutral (Ramakrishnan et al., 2014).

## Baby-Friendly Hospital Initiative

To address low breastfeeding rates, the Baby-Friendly Hospital (BFH) Initiative was established by the World Health Organization (WHO) and the United Nations Children’s Fund in 1991 as a hospital-based intervention. It introduced a set of guidelines for hospitals to protect and support breastfeeding using the Ten Steps to Successful Breastfeeding (TSSB). Among the TSSB is to support the initiation of breastfeeding as soon as possible after birth and maintain breastfeeding and manage common difficulties (Organization, 2017). In order for a hospital to receive a BFH designation, the TSSB must be incorporated into policy and clinical practice requiring curriculum, action plans, quality improvement projects, training, and competency verification (Organization, 2017).

In the U.S., BFH have been associated with higher rates of breastfeeding initiation, duration, and exclusivity. For African Americans specifically, the results of one intervention conducted in four southern states reported that hospitals that increased compliance with the TSSB had decreased racial disparities in breastfeeding initiation and exclusivity. Additionally, there is a dose-response relationship between the number of TSSB steps women are exposed to and improved breastfeeding outcomes, which emphasizes why BFH are important for maternal and child health (Pérez-Escamilla et al., 2016).

Our report aims to examine the association between breastfeeding initiation and having given birth at a BFH in the state of Florida from January 2004 to May 2022. We hypothesized that the odds of breastfeeding initiation would be higher in births that occurred at a hospital that was Baby-Friendly designated compared to non-Baby-Friendly designated hospitals. In addition, we aimed to assess the impact of BFH on breastfeeding initiation specifically in minority groups compared to non-Hispanic White mothers. We hypothesized that breastfeeding initiation would vary by birth at a BFH and maternal race/ethnicity, regardless of covariates, such that infants born at a BFH would have higher initiation rates. Furthermore, we hypothesize that racial/ethnic minority infants born in BFH would still have lower initiation rates than non-Hispanic White infants born in BFH.

## Methods

### Research Design

This study was a retrospective, secondary, exploratory analysis of BFH and birth certificate data in Florida. All study procedures were approved by the State of Florida Institutional Review Boards.

### Setting

Florida is the country’s fourth most populous state, with approximately 21.5 million people (Bureau., 2019). Approximately 18% of Floridians are women of childbearing age (15–44 years old). Nearly a quarter (24.1%) of Florida children live below the Federal Poverty Level compared to the national rate of 16% (U. H. Foundation., 2018b). The racial composition of Florida is 53.2% non-Hispanic White, 16.9% non-Hispanic Black, 26.5% Hispanic, and 3.0% Asian (Bureau., 2019). Similar to other parts of the country, Florida also has large social and health inequities (K. F. Foundation., 2018). For example, 17% of Black individuals and 21% of Hispanic individuals are uninsured versus 12% of non-Hispanic White individuals (K. F. Foundation., 2018). Also, 37.9% of Black individuals and 33.7% of Hispanic individuals are obese versus 28.0% of White individuals (U. H. Foundation., 2018a). In 2018, 48.6% of pregnancy-related deaths in Florida occurred among non-Hispanic Black mothers, while 34.3% occurred among non-Hispanic White mothers (Hernandez, 2020). Furthermore, 5.8 per 1,000 live births in Florida, in 2020 died before reaching their first birthday. This rate is almost doubled in Black infants (F. D. o. Health, 2020).

### Study Population

Approximately 4,009,855 births from January 2004 to May 2022 were included in the dataset. Of these, 3,321,022 were included in the analyses. Births were excluded based on the following criteria: (a) maternal residence was outside the state of Florida, (b) the baby was born prior to 37 weeks of gestation, (c) the baby was admitted to the neonatal intensive care unit, (d) the pregnancy resulted in a multiparous birth, (e) the baby was born with a congenital abnormality, and (f) the birth occurred in a non-hospital setting.

### Data Collection

Data for the analyses were obtained through the Florida Department of Health’s Bureau of Vital Statistics. Where possible, birth certificate data is compiled using the electronic birth record system. Otherwise, births occurring in Florida outside of participating facilities are reported to the county health departments, and then this data is aggregated by the Bureau of Vital Statistics. Variables in this dataset are complex and include items such as demographic and socioeconomic indicators, birth location and outcomes, and complications associated with the birth. County rurality (urban/rural) was obtained from the Florida Department of Economic Opportunity. BFH locations were obtained from the Baby-Friendly USA website that hosts the names, addresses, and month/year of designation of all BFH in the U.S.

### Measurement

The dependent variable of interest, breastfeeding initiation, was obtained from the birth certificate data and was defined as the infant being breastfed during the period between birth and their discharge from the hospital (yes/no). Birth at a BFH was determined by matching the hospital of birth with the designation from Baby-Friendly, USA. Births that occurred at a designated hospital after designation or within one year prior to designation were coded as having occurred at a BFH. We decided to include births within one year prior to designation as a birth at a BFH. Receiving a BFH designation is a complex process that can be lengthy(Organization, 2009). For example, one New York hospital(Goodman & DiFrisco, 2012) reported a 4-year process while a Colorado hospital (Tran, 2017) reported a journey from breastfeeding-friendly to BFH that took 25 years. Ostensibly, a hospital is operating as a BFH for at least a year leading up to official designation.

### Covariates

Relevant covariates for breastfeeding initiation were explored. These included race/ethnicity, maternal education, WIC utilization, prenatal care utilization (Kotelchuck Index), type of delivery (cesarean or vaginal), maternal complications during birth (yes/no), smoking status, marital status at birth (married versus not married), and rural versus urban residence at time of birth. Race and ethnicity were combined into a single variable with the following groups: non-Hispanic White mothers, Hispanic White mothers, non-Hispanic Black mothers, Hispanic Black mothers, other non-Hispanic mothers, and other Hispanic mothers. Maternal education was categorized as: less than high school, high school diploma or equivalent, some college (no degree or Associate’s degree), bachelor’s degree, and master’s or doctorate. Prenatal care utilization followed the Kotelchuck Index with the following categories: inadequate, intermediate, adequate, and adequate plus prenatal care utilization. Smoking status was classified as ever versus never smoker. Furthermore, we included facility codes (codes specific to each hospital and facility) as a covariate to account for any additional hospital-specific effects on breastfeeding initiation.

### Data Analyses

Exploratory data analysis was performed to characterize the sample, stratified by birth at a BFH. A chi-square test for independence was conducted to examine the relationship between birth at a BFH and breastfeeding initiation, and other demographic and clinically relevant variables from the birth certificate data. To investigate if the effect of Baby-Friendly Hospitals on breastfeeding initiation varied by maternal race and ethnicity, we fit a logistic regression model that included the main effects of race and ethnicity, and the exposure (birth at a BFH in the year prior to or anytime following designation). We also fit unadjusted and adjusted models of breastfeeding initiation restricted by maternal race/ethnicity to understand how BFH status influenced breastfeeding initiation by maternal race/ethnicity. Furthermore, to adjust for any additional effects of time, we included time as a sequential variable (beginning at 0 for 2004 through 18 for 2022) to represent the progression of time. Two interaction effects we used in the base models: time*BFH designation, and race/ethnicity*BFH designation. These interaction effects were included in order to determine the linear effects of time and BFH designation on breastfeeding initiation, and the effects of race/ethnicity and BFH designation compared to baseline on breastfeeding initiation. To understand if the interaction of race/ethnicity*BFH designation significantly contributed to the model, we first fit a model with only the main effects and then fit a second model that included the interaction term. The statistical significance of the interaction term was assessed using a Likelihood Ratio Test, comparing the − 2 Log Likelihood values of the two models. An additional multivariate model was fit to adjust for relevant covariates known to affect breastfeeding initiation (see Covariates section). All procedures were conducted in Version 28 of SPSS.

## Results

There are 114 delivery hospitals in Florida, and at the time of analysis, only 25 of them had a BFH designation. Of Florida’s 67 counties, 19 have a BFH, and over a third of the sample resided in a county that did not have a BFH. Table 1 presents characteristics of all births in Florida between 2004 and 2022 that were included in our analyses, by birth at a BFH versus at a non-BFH (during the year prior to, or any year following designation). Overall, only 11% of all births occurred at a BFH, and 84% of mothers initiated breastfeeding. Of the births that occurred at a BFH, 89% initiated breastfeeding. The relationship between breastfeeding initiation and birth at a BFH was significant (*p* < 0.001). Prenatal care utilization, as indicated by the Kotelchuck Index, was relatively high in our sample with 70% of all births having adequate or adequate plus use. However, there was a significant association between prenatal care utilization and birth at a BFH versus a non-BFH (*p* < 0.001). There were also significant relationships between birth at a BFH versus a non-BFH and: maternal race and ethnicity, maternal education, WIC utilization, type of delivery (vaginal versus cesarean), maternal smoking status, county rurality status, and marital status at birth. Speaking to racial and ethnic disparities, while non-Hispanic White mothers made up 44% of the births in our sample, they accounted for 49% of births at a BFH, whereas non-Hispanic Black mothers accounted for nearly 21% of our sample and only 18% of the births at a BFH. Furthermore, among women who delivered at a non-BFH, a larger proportion had an educational attainment of high school diploma/equivalent or less.

**Table 1:** Sociodemographic characteristics

Overall sample	All Births	Birth at a non-BFH	Birth at a BFH	χ^2^	df	*p*
	*n* (%)	*n* (%)	*n* (%)			
	3,321,022 (100)	2,950,467 (89)	370,555 (11)			
Breastfeeding Initiation						
Yes	2,766,178 (84)	2,437,718 (84)	328,460 (89)	6,807.92	1	< 0.001
No	510,612 (16)	470,262 (16)	40,350 (11)			
Maternal Race & Ethnicity						
non-Hispanic White	1,442,997 (44)	1,263,209 (43)	179,788 (49)	5,300.52	5	< 0.001
Hispanic White	951,774 (29)	855,803 (29)	95,971 (26)			
non-Hispanic Black	684,243 (21)	616,421 (21)	67,822 (18)			
Hispanic Black	18,737 (1)	17,344 (1)	1,393 (0)			
Other, non-Hispanic	173,200 (5)	151,396 (5)	21,804 (6)			
Other, Hispanic	22,742 (1)	20,571 (1)	2,171 (1)			
Maternal Education						
Less than High School	500,427 (15)	459,961 (16)	40,466 (11)	6,746.67	4	< 0.001
High School Diploma or equivalent	1,026,947 (31)	902,057 (31)	124,890 (34)			
Some college (no degree or Associate)	922,362 (28)	822,816 (28)	99,546 (27)			
Bachelor’s Degree	576,009 (18)	506,569 (17)	69,440 (19)			
Master’s or Doctorate	27,3205 (8)	239,663 (8)	33,542 (9)			
WIC Utilization						
Yes	1,535,158 (47)	1,374,420 (48)	160,738 (44)	1,749.67	1	< 0.001
No	1,726,723 (53)	1,520,614 (53)	206,109 (56)			
Prenatal Care Utilization (Kotelchuck Index)						
Inadequate use	438,683 (15)	380,149 (14)	58,534 (18)	5,117.63	3	< 0.001
Intermediate use	452,791 (15)	399,471 (15)	53,320 (17)			
Adequate use	1,293,824 (44)	1,156,363 (44)	137,461 (43)			
Adequate plus use	767,260 (26)	695,691 (26)	71,569 (22)			
Type of Delivery						
Vaginal Delivery	2,181,694 (66)	1,926,435 (65)	255,259 (69)	1,902.70	1	< 0.001
Cesarean Section	1,137,926 (34)	1,022,858 (35)	115,068 (31)			
Maternal Complications During Birth						
Yes	142,290 (4)	123,084 (4)	19,206 (5)	821.10	1	< 0.001
No	3,178,732 (96)	2,827,383 (96)	351,349 (95)			
Smoking Status						
Yes, ever	111,189 (3)	96,319 (3)	14,870 (4)	569.77	1	< 0.001
No, never	3,209,833 (97)	2,854,148 (97)	355,685 (96)			
Marital Status						
Yes	1,809,949 (55)	1,605,124 (54)	204,825 (55)	102.34	1	< 0.001
No	1,510,937 (46)	1,345,253 (46)	165,684 (45)			
Rural/Urban Residence						
Rural	157.384 (5)	132,837 (5)	24,511 (7)	3,250.38	1	< 0.001
Urban	3,163,638 (95)	2,817,594 (96)	346,044 (93)			

Table 2 briefly shows adjusted and unadjusted odds ratios of breastfeeding initiation by birth at a BFH for all maternal race/ethnicity groups. Overall, BFH increased breastfeeding initiation for all groups except for Hispanic Black mothers. For example, in the unadjusted model, non-Hispanic White mothers who gave birth at a BFH were 68% more likely to initiate breastfeeding compared to non-Hispanic White mothers who gave birth at a non-BFH (OR = 1.68, CI: 1.66, 1.71, *p* < 0.001). In the adjusted models, the odds of breastfeeding initiation remain higher among mothers who gave birth at a BFH, although remain highest among Hispanic mothers. For instance, Hispanic White mothers who gave birth at a BFH were 26% more likely to initiate breastfeeding (OR = 1.26, CI: 1.22–1.29, *p* < 0.001), whereas non-Hispanic White mothers who gave birth at a BFH were only 13% more likely to initiate breastfeeding (OR = 1.13, CI: 1.11–1.16, *p* < 0.001) compared to Hispanic White and non-Hispanic Black mothers that gave birth at a non-BFH, respectively.

**Table 2:** Unadjusted and adjusted logistic regression results for breastfeeding initiation by birth at a baby-friendly hospital for Race/Ethnicity groups

Race/Ethnicity	OR	[95% CI]	*p*	aOR^a^	[95% CI]	*p*
non-Hispanic White	1.68	[1.66, 1.71]	< 0.001	1.18	[1.16, 1.20]	< 0.001
Hispanic White	1.42	[1.38. 1.45]	< 0.001	1.26	[1.22, 1.29]	< 0.001
non-Hispanic Black	1.52	[1.49, 1.55]	< 0.001	1.13	[1.11, 1.16]	< 0.001
Hispanic Black	1.01	[0.86, 1.19]	0.890	0.87	[0.73, 1.05]	0.147
Other, non-Hispanic	1.55	[1.47, 1.64]	< 0.001	1.10	[1.03, 1.17]	0.004
Other, Hispanic	1.66	[1.41, 1.96]	< 0.001	1.55	[1.28, 1.88]	< 0.001

^a^ Includes covariates: maternal education, WIC utilization, prenatal care utilization (Kotelchuck Index), cesarean delivery, maternal complications during birth, ever smoker (before and/or during pregnancy), rural/urban residence, marital status, facility of birth, and year of birth

In an unadjusted model, the odds of breastfeeding initiation were 57% higher among births at a BFH compared to non-BFH (Table 3; unadjusted model; OR = 1.57, CI: 1.55–1.59, *p* < 0.001). Additionally in Table 3 are the results of the two adjusted logistic regressions. The base model includes only birth at a BFH or not, maternal race/ethnicity, time, and the interactions between birth at a BFH and race/ethnicity and birth at a BFH and time. The multivariate model includes all other clinically relevant covariates. The Likelihood Ratio Test comparing the model with only the main effects to the model that included the interaction term between race/ethnicity and BFH status revealed a significant improvement in model fit (χ²(1) = 194.1, *p* < 0.001). This indicates that the effect of BFHI status on breastfeeding outcomes significantly differs across racial/ethnic categories.

**Table 3:** Logistic regression results for differences in breastfeeding initiation for race by birth at a baby-friendly hospital

	Univariate Model				Base Model^a^				Multivariate Model^b^			
	Coefficient	OR	[95% CI]	*p*	Coefficient	aOR	[95% CI]	*p*	Coefficient	aOR	[95% CI]	*p*
**Birth at a Baby-Friendly Hospital**												
Yes	0.45	1.57	[1.55, 1.59]	< 0.001	0.57	1.77	[1.49, 2.10]	< 0.001	0.61	2.81	[2.31, 3.41]	< 0.001
No	REF	-	-	-	REF	-	-	-	REF	-	-	-
**Race/Ethnicity**												
non-Hispanic White	REF	-	-	-	REF	-	-	-	REF	-	-	-
Hispanic White	0.51	1.66	[1.64, 1.67]	< 0.001	0.52	1.68	[1.66, 1.69]	< 0.001	0.80	1.77	[1.75, 1.79]	< 0.001
non-Hispanic Black	-0.64	0.53	[0.52, 0.53]	< 0.001	-0.64	0.53	[0.52, 0.53]	< 0.001	-0.36	0.63	[0.63, 0.64]	< 0.001
Hispanic Black	0.16	1.17	[1.12, 1.22]	< 0.001	0.12	1.13	[1.08, 1.18]	< 0.001	0.34	1.24	[1.78, 1.30]	< 0.001
Other, non-Hispanic	0.45	1.56	[1.54, 1.59]	< 0.001	0.42	1.53	[1.50, 1.55]	< 0.001	0.31	1.23	[1.21, 1.26]	< 0.001
Other, Hispanic	0.33	1.39	[1.33, 1.44]	< 0.001	0.27	1.31	[1.26, 1.37]	< 0.001	0.53	1.60	[1.53, 1.68]	< 0.001
**Time**												
Year	0.05	1.06	[1.05, 1.06]	< 0.001	0.05	1.05	[1.05, 1.06]	< 0.001	0.05	1.05	[1.05, 1.05]	< 0.001
Interaction Terms												
Birth at BFH * Race/Ethnicity												
Any race/ethnicity * Birth non-BFH	REF	-	-	-	REF	-	-	-	REF	-	-	-
Non-Hispanic White * Birth BFH	0.53	1.69	[1.66, 1.72]	< 0.001	-0.02	0.98	[0.83, 1.15]	0.789	-0.11	0.84	[0.70, 1.01]	0.063
Hispanic White * Birth BFH	0.88	2.42	[2.36, 2.48]	< 0.001	-0.21	0.81	[0.69, 0.96]	0.013	-0.28	0.74	[0.62, 0.89]	0.002
non-Hispanic Black * Birth BFH	-0.20	0.82	[0.80, 0.84]	< 0.001	-0.14	0.87	[0.74, 1.03]	0.105	-0.24	0.73	[0.61, 0.88]	< 0.001
Hispanic Black * Birth BFH	0.23	1.26	[1.08, 1.47]	0.004	-0.49	0.62	[0.49, 0.78]	< 0.001	-0.55	0.64	[0.49, 0.82]	< 0.001
Other, non-Hispanic * Birth BFH	0.90	2.46	[2.34, 2.59]	< 0.001	-0.09	0.92	[0.77, 1.09]	0.312	-0.27	0.71	[0.58, 0.86]	< 0.001
Other, Hispanic * Birth BFH^c^	0.89	2.35	[2.01, 2.76]	< 0.001	Na	Na	Na	Na	Na	Na	Na	Na
Birth at BFH * Time	0.03	1.03	[1.03, 1.03]	< 0.001	-0.03	0.98	[0.97, 0.98]	< 0.001	-0.02	0.95	[0.94, 0.95]	< 0.001

^a^ Base model only includes variables specified in the table

^b^ Includes additional covariates: maternal education, WIC utilization, prenatal care utilization (Kotelchuck Index), cesarean delivery, maternal complications during birth, ever smoker (before and/or during pregnancy), rural/urban residence, marital status, and facility of birth

^c^ Model was reduced to exclude these births due to redundancies (Na)

In the base model, mothers who gave birth at a BFH were 77% more likely to report initiating breastfeeding compared to mothers who gave birth at a non-BFH (OR = 1.77, CI: 1.49–2.10, *p* < 0.001). When accounting for all other covariates, mothers who gave birth at a BFH were 181% more likely to initiate breastfeeding compared to mothers who gave birth at a non-BFH (OR = 2.81, CI: 2.31–3.41, *p* < 0.001). There were some notable differences in breastfeeding initiation by maternal race/ethnicity. Considering the main effect of maternal race/ethnicity, non-Hispanic Black mothers were the only group that had lower odds (37% less likely) of breastfeeding initiation compared to non-Hispanic White mothers (OR = 0.63, CI: 0.64–0.64, *p* < 0.001). Examining the interaction between birth at a BFH and race/ethnicity in the adjusted models, all minority race/ethnicity groups who gave birth at a BFH were less likely to initiate breastfeeding compared to mothers of any race that gave birth at a non-BFH. For example, non-Hispanic Black mothers who gave birth at a BFH were 27% less likely to initiate breastfeeding compared to mothers of any race/ethnicity who gave birth at a BFH (OR = 0.73, CI: 0.61–0.88, *p* < 0.001) in the fully adjusted model. Similar results were observed for two maternal race/ethnicity groups (Hispanic White mothers, and Hispanic Black mothers) in the base model, and three other maternal race/ethnicity groups (Hispanic White, Hispanic Black, and other non-Hispanic mothers) in the fully adjusted model.

## Discussion

Our report examines the impact of BFH on breastfeeding initiation among different racial and ethnic groups in the state of Florida. Approximately 23% of the hospitals in Florida have a BFH designation, however, only 11% of births between 2004 and May of 2022 occurred at a BFH, indicating a need to further explore the barriers that may preclude mothers from giving birth at a BFH. Similar to existing literature, we found that giving birth at a BFH is associated with greater odds of breastfeeding initiation. However, we also found significantly lower odds of breastfeeding initiation among most minority mothers who gave birth at a BFH compared to any mothers who gave birth at a non-BFH. This is to say that although women giving birth at a BFH have greater breastfeeding initiation rates than their racial/ethnic counterparts at non-BFH, the impact of BFH is weaker for minority women. Not only do breastfeeding initiation disparities persist at BFH, but they may actually be greater than in non-BFH.

Our results contribute to evidence of the significant disparities in maternal care and outcomes among racial and ethnic minorities in the U.S., particularly in Florida. As seen in our analysis, Hispanic mothers, regardless of race, had higher odds of initiating breastfeeding compared to NH White mothers than any of the other racial/ethnic groups examined in this paper. This is consistent with the existing literature. Overall, Hispanic breastfeeding initiation rates are comparable to NH White mothers, but rates lag in breastfeeding duration (Louis-Jacques et al., 2017). Typically, when Hispanic breastfeeding rates are disaggregated by language preference, acculturation, and country of origin, there is great variation (Gill, 2009). For example, foreign-born Hispanic women are more likely to initiate and continue breastfeeding than US-born Hispanics (Gill, 2009). One novel approach relevant to our findings is to disaggregate Hispanics by race (Lebron et al., 2023). We found that, although Hispanic Black mothers initiate breastfeeding at higher rates than NH White women, they do not outpace at the same rate as Hispanic White mothers. Furthermore, in the multivariate model, Hispanic Black mothers were significantly less likely to initiate breastfeeding if giving birth at BFH than NH White mothers. This was not a significant finding for Hispanic White mothers. This finding further supports systemic inequities of race and ethnicity.

While some studies have found that BFHs have worked to correct breastfeeding disparities (Merewood et al., 2007, 2019), our findings suggest otherwise. Racial and ethnic disparities in breastfeeding initiation and duration are rooted in a wide range of interrelated historical, cultural, social, economic, political, and psychosocial factors (Louis-Jacques et al., 2017). Yet, Hemingway et al. (2021) rightly point out that “focusing on individual factors rather than the systemic inequities such as racism in the social and health care infrastructure leads to ineffective interventions that are centered on external factors beyond the individual’s control.” Studies have consistently found differences in care by race/ethnicity. For instance, a review of missed care and equitable breastfeeding support found that Spanish-speaking parents were more likely to be given formula at discharge, African American women reported not receiving breastfeeding help, and Hispanic parents were more likely to receive pacifiers or artificial nipples (Gallagher & McKechnie, 2024). Interviews with International Board Certified Lactation Consultants (IBCLCs) by Thomas (2018) revealed systemic race-based discrimination. This included presumptions that women of color would not breastfeed, fewer lactation referrals, deprioritization in workflow, and overt racist comments. These discriminatory practices likely contribute to lower breastfeeding rates among minority mothers. Additionally, a study of 283 U.S. birthing facilities revealed that only 9% had a specific plan to support breastfeeding among women of color (Gonzalez-Nahm & Benjamin-Neelon, 2023). Surprisingly, having a BFH designation did not correlate with such a plan. Many facilities reported a “color-blind” approach, treating all women the same regardless of race or ethnicity. While this may seem equitable, it fails to address the specific needs and barriers faced by women of color and may perpetuate behaviors that contribute to disparities (Gonzalez-Nahm & Benjamin-Neelon, 2023).

It is important to note that while in this study our focus is on BFH, the hospitals are only one aspect of lactation support. In one review of the impact of BFH (Munn et al., 2016), researchers found that step 3, prenatal education, and step 10, postnatal breastfeeding support are the most difficult steps to implement. This is especially important because researchers have found that Black women are less likely to attend prenatal education classes and mothers who attended childbirth classes were 75% more likely to initiate breastfeeding than those who did not (Lu et al., 2003). As for postnatal breastfeeding support, the Affordable Care Act demands that private insurers and expanded Medicaid plans provide “comprehensive lactation support and counseling by a trained provider during pregnancy and/or in the postpartum period”. However, the interpretation of what constitutes a trained provider has been left to insurers; this combined with the 20 states without Medicaid expansions (including Florida) has left a gap for women in need of critical lactation support (Wouk et al., 2017). Furthermore, women who engaged in *both* prenatal education and ongoing post-discharge support had greater breastfeeding duration than women who participated in prenatal intervention alone (Schreck et al., 2017).

The importance of culturally specific interventions and multicultural sensitivity training for healthcare staff should be further explored. Implementing the Baby-Friendly initiative alone may not be enough to close the racial gap in exclusive breastfeeding rates at hospital discharge (Hemingway et al., 2021; Louis-Jacques et al., 2017). Research shows that interventions to reduce disparities are more successful when healthcare staff are aware of these disparities and prepared to address them (Equity, 2024). In one intervention designed to implement BFH initiatives, highlighting racial disparities to staff proved the most impactful test of change (Knutson & Butler, 2022). Furthermore, studies have shown that patient-provider race concordance creates a positive rapport and establishes trust (Petit et al., 2021). This highlights the need for a diverse healthcare workforce that can offer culturally competent care and support to all mothers, regardless of their racial or ethnic background (Gonzalez-Nahm & Benjamin-Neelon, 2023; Hemingway et al., 2021).

### Limitations

Although our study makes a significant contribution to the literature, it must be considered in light of several limitations. Breastfeeding initiation data might be misclassified. Although a comparison of birth certificates to medical records across 8 hospitals in 2 states found high sensitivity of breastfeeding initiation, moderate false discovery rates suggest that discrepancies might exist between medical records and birth certificates (Martin et al., 2013). However, overall rates are generally consistent with other national data sources. We did not include the presence of other breastfeeding organizations of support such as La Leche League. Further, we only examined the association between BFH and breastfeeding initiation. While breastfeeding initiation is a key breastfeeding indicator, national and global organizations such as the AAP and the WHO recommend exclusive breastfeeding for the first 6 months of life. However, breastfeeding exclusivity is not a measure captured in birth certificate data. Finally, the comparison group included non-BFH as well as BFH one year prior to their designation. Although those BFH tend to be breastfeeding-friendly long before their designation, there are clear differences between BFH and those without the designation. The policies and training necessary for this are challenging and complicated even for a breastfeeding-friendly hospital (Tran, 2017). As such, we feel that this method of analysis was necessary to both include those hospitals operating as a BFH and those not. Lastly, we did not control for baseline differences in breastfeeding initiation between hopsital times. While the observed effect sizes may reflect both the impact of BFH status and any pre-existing differences in breastfeeding initiation rates, the results still provide valuable insights into the overall relationship. However, this approach allowed us to capture the cumulative effect of BFH policies as they interact with existing hospital practices, offering insights into the full scope of their impact on breastfeeding initiation.

## Conclusions

Our study found that the relationship between breastfeeding initiation and birth at a BFH varied by race/ethnicity. Although BFH has an impact on breastfeeding initiation, a lack of culturally competent implementation may work to widen the disparities in breastfeeding leading to associated undesirable health outcomes.

## Data Availability

The data used in this study was made available by the Florida Department of Health.

## Code Availability

Code is available upon request.
